# Lipid mediators in diabetic nephropathy

**DOI:** 10.1186/1755-1536-7-12

**Published:** 2014-09-03

**Authors:** Swayam Prakash Srivastava, Sen Shi, Daisuke Koya, Keizo Kanasaki

**Affiliations:** 1 Department of Diabetology & Endocrinology, Kanazawa Medical University, Uchinada, Ishikawa 920-0293, Japan; 2 Division of Anticipatory Molecular Food Science and Technology, Kanazawa Medical University, Uchinada, Ishikawa 920-0293, Japan

**Keywords:** Diabetic nephropathy (DN), Protein kinase Cs (PKCs), Diacylglycerol (DAG), Glyceroshingolpids, Antidyslipidaemic drugs and microRNAs (miRs)

## Abstract

The implications of lipid lowering drugs in the treatment of diabetic nephropathy have been considered. At the same time, the clinical efficacy of lipid lowering drugs has resulted in improvement in the cardiovascular functions of chronic kidney disease (CKD) patients with or without diabetes, but no remarkable improvement has been observed in the kidney outcome. Earlier lipid mediators have been shown to cause accumulative effects in diabetic nephropathy (DN). Here, we attempt to analyze the involvement of lipid mediators in DN. The hyperglycemia-induced overproduction of diacyglycerol (DAG) is one of the causes for the activation of protein kinase C (PKCs), which is responsible for the activation of pathways, including the production of VEGF, TGFβ1, PAI-1, NADPH oxidases, and NFҟB signaling, accelerating the development of DN. Additionally, current studies on the role of ceramide are one of the major fields of study in DN. Researchers have reported excessive ceramide formation in the pathobiological conditions of DN. There is less report on the effect of lipid lowering drugs on the reduction of PKC activation and ceramide synthesis. Regulating PKC activation and ceramide biosynthesis could be a protective measure in the therapeutic potential of DN. Lipid lowering drugs also upregulate anti-fibrotic microRNAs, which could hint at the effects of lipid lowering drugs in DN.

## Review

### Introduction

Abnormalities in the lipoprotein metabolism are associated with patients with diabetic nephropathy (DN) [1]. The lipoprotein abnormalities include a higher level of very low-density lipoproteins (VLDL-c), low-density lipoprotein-cholesterol (LDL-c) and a suppressed level of HDL-cholesterol (HDL-c). Researchers have reported that lipoprotein abnormalities are associated with the development of glomeruloscerosis, a process that may share similarities with atherosclerosis [2]. At the same time, clinical studies have shown that in patients with DN, lipid-lowering drugs could be associated with a reduction in the proteinuria but not with a remarkable improvement in the kidney outcome [3,4]. A high level of LDL-cholesterol has been found to predict the development of microalbuminuria in type 1 diabetes [5]. Clinical studies on the association between hyperlipidemia and renal dysfunction in patients with DN are difficult to perform due to the complex interrelationship between serum lipids, blood glucose, and proteinuria. Watts *et al.* found that the development of microalbuminuria in a 10-year follow-up was closely related to the baseline serum cholesterol and LDL cholesterol levels [6] in a study on 53 normoalbuminuric type 1 diabetic patients. Parving *et al*. also found a close association between the serum cholesterol level and progression of renal dysfunction in a 10-year prospective study in type 1 diabetic patients with nephropathy [7]. The baseline level of serum cholesterol was also found to be the independent risk factor for the development of DN in studies of patients with type 2 diabetes [8]. In epidemiologic studies of individuals with glomerular disease or DN, hyperlipidemia predicted a faster loss of kidney function [9]. Krolewski *et al*. [10] evaluated predictors of fast progression (creatinine after 3 years of follow-up/baseline creatinine 41.5) in 424 individuals with type 1 diabetes and proteinuria. The proportion of individuals with fast progression was 29% in those with a baseline total cholesterol of 180 mg/dl, 24% for 180 to 219 mg/dl, 38% for 260 to 299 mg/dl, and 48% for >300 mg/dl [10,11]. The findings persisted after controlling for blood pressure; however, the studies did not control for the degree of proteinuria. In the RENAAL study, the baseline total cholesterol (HR (hazard ratio) 1.96 per 100 mg/dl ml higher) and LDL cholesterol (HR 1.47 per 50 mg/dl higher) were associated with a higher risk of progression to end-stage renal disease [12]. It is unclear from the report whether they controlled for baseline proteinuria. The issue of whether the prior studies controlled for proteinuria is relevant given the strong correlation between the serum cholesterol levels and renal albumin clearance [13].

The beneficial effects of statins on DN progression also remain unclear due to the lack of prospective, randomized intervention studies. Early studies have demonstrated that treatment with the HMG CoA reductase inhibitor pravastatin decreases albuminuria in patients with type 2 diabetes [14,15]. However, later studies showed the beneficial effects on albuminuria with HMG CoA reductase inhibitors (simvastatin) in both type 1 or type 2 patients [16,17]. These studies involved only a small number of patients, and the follow-up duration was short, making it inadequate for assessing the clinical effects of lipid control in DN.

### Lipid mediators as modulators of renal physiology

The families of prostaglandins, leukotrienes, and related compounds are called eicosanoids and are derived from 20-carbon polyunsaturated fatty acids (dihomo-γ-linolenic acid, arachidonic acid, or eicosapentaenoic acid [EPA]). These compounds have extremely widespread and diverse biological effects, some of which may be of great importance in the pathophysiology of renal disease. The prostaglandins PGE2 and PGI2 help maintain renal blood flow and glomerular filtration in clinical conditions that are associated with renal functions, and they are generally considered beneficial. PGI3 has been reported to have a similar efficacy to PGI2. Conversely, thromboxane A2 (TXA2) decreases renal blood flow and glomerular filtration [18]. Remarkable increases in the renal production of prostaglandins and/or TXB2 have been reported in humans with chronic renal disease and various animal models of renal disease [19]. Pharmacological or dietary intervention to alter these changes in eicosanoids may provide a means of modifying the disease process. The inclusion of fat sources rich in EPA (for example, marine fish oils) would be expected to decrease the production of dienoic prostaglandins and increase that of the trienoic series [20]. Dietary fish oil supplementation is beneficial in some animal models of renal disease that involve an immune component, for example, murine lupus [21]. A remarkable decrease in the production of dienoic eicosanoids and the increased production of PGE3 (although levels were much lower than those of the dienoic compounds) were noted with fish oil supplementation in one of these studies [21]. It is of interest that a diet high in linoleic acid was beneficial in another subtotal nephrectomy study in rats, although such kind of beneficial effect of linoleic acid was observed in the absence of changes in the urinary excretion of dienoic eicosanoids [22]. Further studies are needed to better evaluate the potential for using polyunsaturated fatty acids in naturally occurring diseases in other species.

### Role of PKC activation in DN

**Figure 1:**
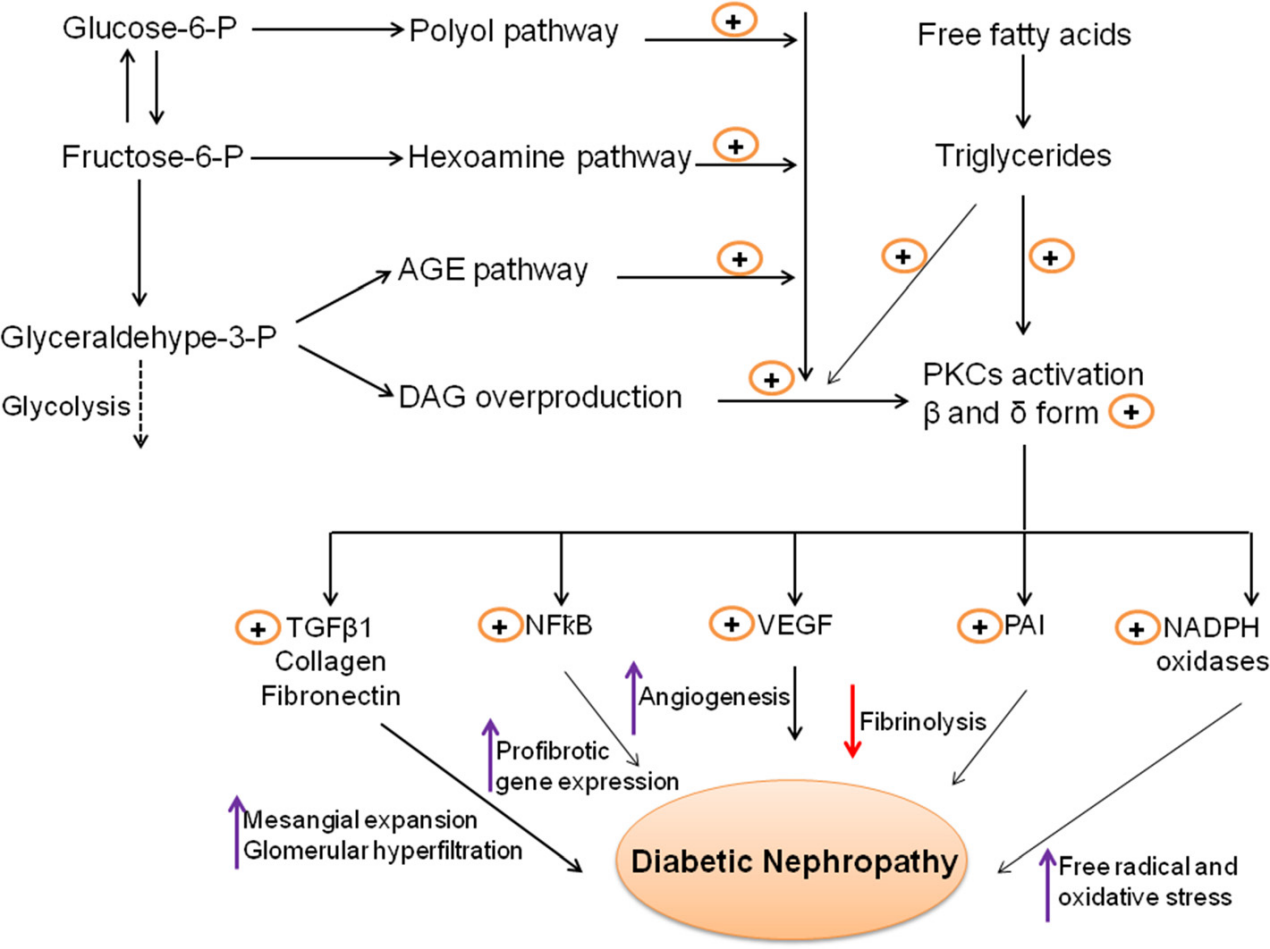
**Multiple sources of diacylglycerol (DAG), and DAG-mediated PKC activation could contribute to renal fibrosis.** Overproduction of DAG causes the activation of PKC, leading to the initiation and development of diabetic nephropathy (DN). PKC (β and δ form) primarily upregulates the NFҟB gene, TGFβ1 action, VEGF, PAI, and NADHP oxidases pathway. Increased TGFβ1, collagen, fibronectin enhanced mesangial expansion, activated NFҟB induce profibrotic gene expression, and activated VEGF cause enhanced angiogenesis, and upregulated PAI reduces fibrinolysis, whereas increased NADPH oxidase provides oxidative stress to renal cells; all of these factors could contribute to the development of renal fibrosis.

Protein kinase C (PKC) is involved in signal transduction pathways, cell proliferation, differentiation, cell cycle, and apoptosis. However, the role of PKC in kidney disease has not yet been fully defined. The PKC activation induced by hyperglycemia is likely due to the increase in the 1,2-diacylglycerol (DAG) levels, a physiological activator of PKC, although other co-factors such as phosphatidylserine (PS) or phorbolester (PE) are also known stimulants. Inactivation of the PKC isoforms has been found to counteract many hyperglycemia associated vascular dysfunctions in the kidney [23]. The PKC family has 13 isoforms, which have been classified into three subfamilies based on their mode of activation. The group of classical or conventional PKC (cPKC) consists of the α, βI, βII, and γ isoforms, all of which depend on calcium (Ca^++^), DAG or its analogue phorbol 12-myristate 13-acetate and, in most cases, phosphatidyl serine (PS) for activation. The isoforms that are independent of Ca^++^ but require DAG and PS are classified as the new PKC (nPKC), and include the subtypes δ, ϵ, η, and θ. The third group of isoforms is that of the atypical PKC (aPKC) ξ, ι/λ, ν, and μ. Their activation does not require Ca ^++^ or DAG; they only require PS. Figure 1 depicts the different sources of DAG production and effects on PKC activation. The activation of PKC (β and δ form) leads to the initiation of many pathological processes responsible for the development of DN. The pathological processes include production of VEGF, TGFβ1, PAI-1, and NADPH oxidases and activation of the NFҟB signaling pathway. Once activated, PKC can transmit signals to the nucleus via different signal transduction pathways. For example, the mitogen-activated protein kinase (MAPK) cascade may be activated. Such diverse functions as cell proliferation, differentiation and cell death are outcomes of particular MAPK members: Raf-1 can be activated by Ras in some cases after crosstalk with PKC; activated extracellular signal regulated kinases can in turn activate transcription factors such as myc, myb, fos, and jun, thereby enabling the expression of genes encoding enzymes required for key metabolic functions, such as cell proliferation and invasion; and the JNK pathway often initiates cell death, which, in some cases, is stimulated by PKC isoforms, as recently reviewed by Li and Gobe [24].

Koya *et al*. reviewed the activation of PKC-associated development of diabetic complication [25]. The inhibition of PKCβ, a common molecule in diabetes-related renal and vascular injury, holds promise as a novel strategy for improving micro- and macro-vascular complication outcomes in diabetes [26]. Key mechanisms in the pathogenesis of vascular dysfunction in DN are ROS formation followed by the activation of specific isoforms of PKC. The *de novo* formation of DAG during hyperglycemia activates PKC. Acute hyperglycemia caused the activation of PKC, which could also be associated with rapid endothelial impairment in DN. In the STZ rat model, blockade of the PKCβII isoform with LY333531 prior to hyperglycemia protected nitric oxide formation within the arteriolar wall [27]. Additionally, the increased secretion of inflammatory molecules as a result of hyperglycemia induced PKCs activation, which is involved in oxidative stress and associated with vascular dysfunction in DN. The findings from various studies support an association between the increased secretion of inflammatory molecules, such as cytokines, growth factors, and metalloproteinases, and the development of DN [28,29].

### Ceramide in DN

**Figure 2:**
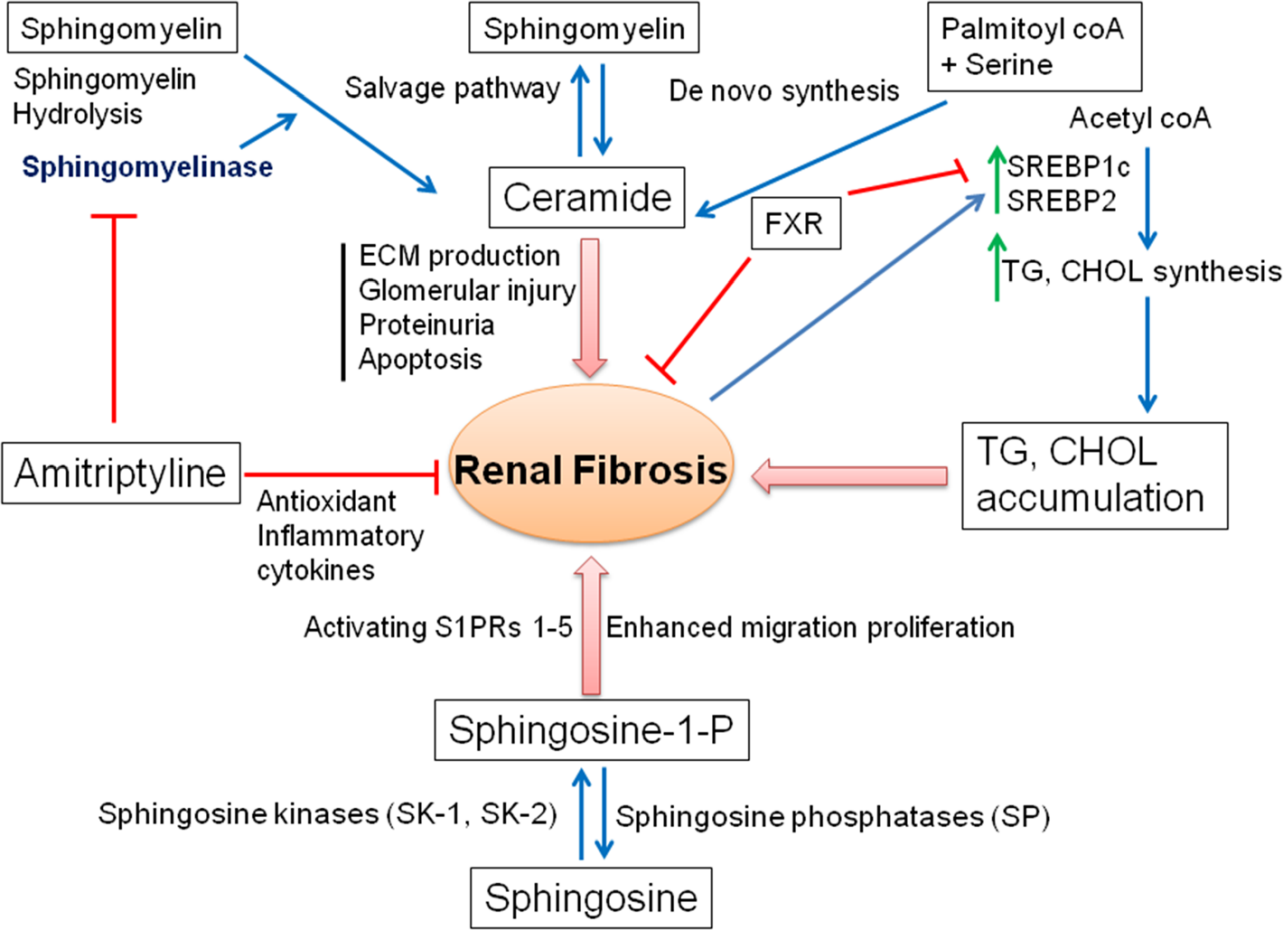
**Schematic presentation of ceramide synthesis and its involvement in renal fibrosis.** Ceramide is synthesized mainly by three processes: *de novo* synthesis, the salvage pathway, and sphingomyelin hydrolysis. Ceramide could cause renal fibrosis by enhancing ECM production, glomelular injury, proteinuria, and apoptosis. Triglycerides (TGs) and cholesterol (CHOL) accumulation in the kidney could also contribute to the development of renal fibrosis. At the same time, sphingosine kinase could contribute to renal fibrosis by enhancing cell migration and proliferation. The renoprotective activity of amitriptyline is due to its ability to inhibit the sphingomyelinase enzyme and thereby inhibit ceramide synthesis and to protect against renal fibrosis.

Ceramides are highly abundant in the kidney, and they regulate various cellular processes, including cell proliferation, apoptosis, inflammation, and cellular signaling. Ceramide consists of N-acetylated (14 to 26 carbons) sphingosine (16 to 18 carbons). Figure 2 depicts the role of sphingomyelin in the development of kidney disease. The role of sphingomyelin and ceramide in renal physiology was previously reviewed by Hao and Breyer [30,31]. Ceramide is mainly produced from the hydrolysis of sphingomyelin, which is catalyzed by sphingomyelinase [32]. Ceramide can also been generated through the condensation of sphingosine or sphinganine and fatty acyl-CoA by ceramide synthase [32]. The direct targets of ceramide are ceramide-activated protein phosphatase, ceramide-activated protein kinase, and protein kinase Cξ. Previous studies have shown that ceramide is involved in the pathogenesis of acute kidney injury caused by ischemic reperfusion, toxic insults, and oxidative stress [33]. In a normal mouse kidney cortex, C24, C22, and C16 ceramides have been identified [34]. Ischemia/reperfusion or nephrotoxic injury (glycerol-mediated myohemoglobinuria, radiocontrast) causes a transient reduction in the renal ceramide levels, which is followed by a two- to three-fold increase in the ceramide levels [35]. The increased ceramide after renal injury is not associated with the enhanced hydrolysis of sphingomyelin because sphingomyelinase expression was not increased (instead, it is decreased) in the experiments [33]. In contrast, hypoxia reoxygenation or radiocontrast-induced renal tubular epithelial cell injury is attenuated by the ceramide synthase inhibitor, fumonisin B1, suggesting that increased ceramide synthase activity is responsible for the increased ceramide generation, leading to apoptotic change of the renal epithelial cells [35,36]. Achar *et al.* found that the tricyclic antidepressant amitriptyline inhibited inflammation, myofibroblast formation, and several other indices of renal fibrosis in ureterally obstructed (UUO) mice [37]. Additionally, there were significant reductions in the expression of α-smooth muscle actin (α-SMA), osteopontin, transforming growth factor-β (TGFβ), monocyte chemotactic protein-1 (MCP-1), and intercellular adhesion molecule-1 due to amitriptyline treatment in UUO mice [38]. These pleiotropic antidepressant agents have antifibrotic effects in the kidney and other organ systems. Antidepressants accomplish reduction in kidney fibrosis by several mechanistic possibilities, such as by reducing inflammation, the number of natural killer cells, inflammatory cytokines, chemokines, H-1 receptor, and acid sphingomyelinase activity [39] or increasing anti-oxidant activity in the UUO mice [37]. Amitriptyline can degrade acid sphingomyelinase, an enzyme contributing to ceramide formation, and degradation of acid sphingomyelinase could be helpful in the treatment of DN. Achar *et al.*[40] studied the renoprotective effect of amitriptyline in UUO mice. The beneficial effect of amitriptyline in the UUO study by Achar *et al.*[40] could be an inhibition of acid sphingomyelinase that, in turn, inhibits tubular-cell apoptosis and is interstitial in inflammation. A gene knock out experiment to study acid sphingomyelinase would helpful for understanding the protective effect of amitriptyline. Based on the aforementioned reports, we conclude that such a pleiotropic effect of the tricyclic anti-depressant would be a therapeutic challenge in DN.

### Antidyslipidemic drugs in DN

As in the previous Japanese atherosclerosis society (JAS) guidelines of 2007, an LDL goal of <120 mg/dl has been set for the high-risk primary prevention patients, and a goal of <100 mg/dl is recommended for secondary prevention patients. Therefore, physicians need to help secondary prevention patients in particular achieve the current LDL-c targets using standard lipid management strategies, which can be followed by a more stringent treatment approach. The effect of statins in renal disease have been discussed in detail in many reviews [41,42]. Table 1 summarizes the beneficial effects of lipid lowering drugs on renal outcomes. It is interesting that some of the beneficial effects of statins can be independent of the cholesterol reduction [43]. Different classes of statins can provide renoprotective activity. The potential renal protective effects of statins include protection against oxidative stress, amelioration of endothelial function through nitric oxide (NO) production and changes in blood rheology. The difference in the renal protective effects of different class of statins are not clearly known in DN lipid-related mechanisms, but this could be due to the statins different pharmacological, pharmacokinetic properties, bioavailability, and distribution in the tissues. A randomized comparison of pitavastatin and pravastatin treatment on the reduction of urinary albumin in patients with type 2 diabetic nephropathy was studied [44]. Pitavastatin was more effective than pravastatin in reducing albuminuria in type 2 diabetic patients with early-stage diabetic nephropathy [44]. Rosuvastatin administration reduced albuminuria, oxidative stress, and the serum cystatin C levels, independent of blood pressure or lipid levels in DN patients [45]. Protection Against Nephropathy in Diabetes with Atorvastatin (PANDA), a randomized, double-blind placebo-controlled trial of high- *versus* low-dose atorvastatin (1) studies, reported no significant difference in the renal functions between those taking high or low doses of atorvastatin over 2 years [46]. Another report on the effects of atorvastatin on kidney outcomes and cardiovascular disease in patients with diabetes, an analysis from the Collaborative Atorvastatin Diabetes Study (CARDS), revealed a beneficial effect of atorvastatin on the estimated glomerular filtration rate (eGFR), particularly in patients with albuminuria [47]. Atorvastatin was effective at decreasing CVD in those with or without a moderately decreased eGFR and achieved a high absolute benefit [47].

**Table 1:** Effect of lipid lowering drugs on the renal outcome

**Drugs**	**Effect on renal outcome**	**References**
Statins and Fenofibrate	Reduction in urinary albumin	[3,4]
Atorvastatins with combine therapy with glycaemic control	Inhibit cardiovascular and renal end points by 50% in DN patients	[46]
Pitavastatin and Pravastatin	Reduction in urinary albumin in DN patients	[44]
	Pitavastatin is more effective than Pravastatin	
Rosuvastatin	Reduction in albuminuria, oxidative stress, and serum cystatin C levels in DN patients	[45]
Atorvastatin	Not remarkable difference in renal functions between those taking high or low dose atorvastatin over 2 years	[46]
Atorvastatin	Atorvastatin was effective at decreasing CVD in those with and without a moderately decreased eGFR	[47]
Fenofibrate	Not remarkable difference in GFR, Provide reno-protection in moderate renal impairment patients	[56,58]
Fenofibrate	Inhibit risk of albuminuria in patient with diabetes	[59]
Fenofibrate	Acute change in creatinine not associated with adverse effect on renal outcome	[56,57]
Simvastatin (and simvastatin + ezetimibe)	Does not produce remarkable reductions in measures of renal disease progression	[61-63]

In an elegant study by Zoja *et al. *[48], the authors reported that a combined angiotensin converting enzyme inhibitor and statin therapy had a remarkable anti-proteinuric effect with a significant improvement in the renal function in a severe rat model of passive Heymann nephritis (PHN). The drug combination limited glomerulosclerosis, tubular damage, and interstitial inflammation compared with placebo or drugs alone.

*In vitro* studies have clearly established that statins influence important intracellular pathways that are involved in the inflammatory and fibrogenic responses [43]. Statins also inhibit the proliferation of cultured mesangial cells and renal epithelial tubular cells through their ability to suppress the formation of intermediate metabolites of the mevalonate pathway, particularly the nonsterol isoprenoids, which seem to be essential in cell replication [49]. Multifactorial strategies that combined glycemic therapy, lipid lowering, blood pressure (BP) control, and aspirin reduced both the cardiovascular and renal end points by 50% in patients with diabetes and microalbuminuria [50].

Recently, lipid lowering agents in chronic kidney disease were reviewed by researchers, which was of great interest for us [51]. Statins remarkably improve cardiovascular disease (CVD) and the associated mortality in patients with CKD both with and without diabetes who do not require dialysis [52,3]. Statin treatment protects from the injuries caused by the DAG-Activated PKC activation in a variety of ways. These pleotropic effects of statins include improvement in the endothelial function, reduction in the oxidative stress, reduction in the TGFβ1 level, decline in the level of inflammatory cytokines and sensitization of the Akt/PI3 kinase pathways, inducing the GLUT 4 translocation reviewed by Danesh and Kanwar [53]. The effect of lipid lowering drugs on ceramide formation is not well known.

Fibrate is another class of lipid lowering drugs to treat hypertriglyceridaemia with residual elevation of non-HDL cholesterol. However, the role of fibrates in patients with CKD has yet to be determined [54]. Investigators in the Fenofibrate Intervention and Event Lowering in Diabetes (FIELD) study [55] randomly allocated [56] 795 participants with type 2 diabetes mellitus to either fenofibrate (200 mg daily) or placebo, with a median follow-up period of 5 years. In this study, fenofibrate treatment reduced the total cardiovascular events (HR 0.89, 95% CI 0.80-0.99) [55]. This benefit did not differ significantly between groups that had different eGFRs. In another report, the researchers summarized that fenofibrate should be considered as an additional therapeutic option, along with conventional risk-factor management, to further reduce CVD events and mortality and provide reno-protection to patients with diabetes and to those with moderate renal impairment [4]. In a meta-analysis, fibrates reduced the risk of albuminuria progression in patients with diabetes and reduced the risk of major cardiovascular events and cardiovascular death (but not all-cause mortality) in patients with an estimated glomerular filtration rate of 30 to 59.9 mL/min/1.73 m^2^ [57]. Fibrates also increase the serum creatinine level after therapy initiation, but this effect could be a response to the blunting of creatinine secretion rather than to true renal injury [58]. In a post-trial analysis of the FIELD and ACCORD datasets, acute changes in the creatinine level with fibrate therapy were not associated with adverse effects on the major clinical renal outcomes.

Researchers introduced a Study of Heart and Renal Protection (SHARP) study, was a double-blind, placebo-controlled trial that aimed to assess the safety and efficacy of reducing LDL cholesterol in more than 9,000 patients with chronic kidney disease (approximately 3,000 of whom were on dialysis at randomization) [59,60]. Among the CKD patients, the most common recorded cause of renal disease was glomerulonephritis (19%), diabetic nephropathy (15%) and hypertensive or renovascular disease (20%) [61]. Among the 6,000 patients who were not receiving dialysis at randomization, the average eGFR was 26.5 (SD 13.0 mL/min/1.73 m^2^, approximately two-fifths of the patients who were not on dialysis had kidney disease outcome quality initiative stage 3 disease. Among the non-dialysis patients who had a measurement of the urinary albumin to creatinine ratio (ACR), approximately one-fifth had a value in the reference range (ACR <30 mg/g), two-fifths had microalbuminuria (ACR >30 mg/g), and two-fifths had macroalbumiuria (>300 mg/g) [61]. In high-risk populations (such as patients with diabetes), observational studies have shown that there is an approximately log-linear relationship between the risk of death from cardiovascular disease and blood cholesterol, but an analogous association has not been demonstrated in with CKD. In the study, associations between blood cholesterol and cardiovascular mortality were found on a logarithmic scale, among the screenees in the Multiple Risk Factor Intervention Trial (MRFIT) with diabetes (*N* = 5000) and without diabetes (*N* = 340,000) [59]. The study authors also aimed to assess whether lowering LDL cholesterol reduced the rate of loss of renal function in people with CKD, with or without diabetes, who had not commenced dialysis treatment [59,60]. Patients were randomly assigned to receive simvastatin 20 mg plus ezetimibe 10 mg daily *versus* matching placebo. SHARP concluded that approximately a quarter of all heart attacks, strokes, and operations to open blocked arteries could be avoided in people with CKD by using a combination of ezetimibe and simvastatin to lower the blood cholesterol levels [59]. Of the approximately 6,000 patients who were not on dialysis at randomization, allocation to simvastatin plus ezetimibe did not produce significant reduction in any of the prespecified measures of renal disease progression. End stage renal disease is defined as the commencement of maintenance dialysis or transplantation; end stage renal disease or death; and end stage renal disease or doubling of baseline creatinine [61].

### MicroRNAs as new regulatory molecules targeted by lipid lowering drugs

**Figure 3:**
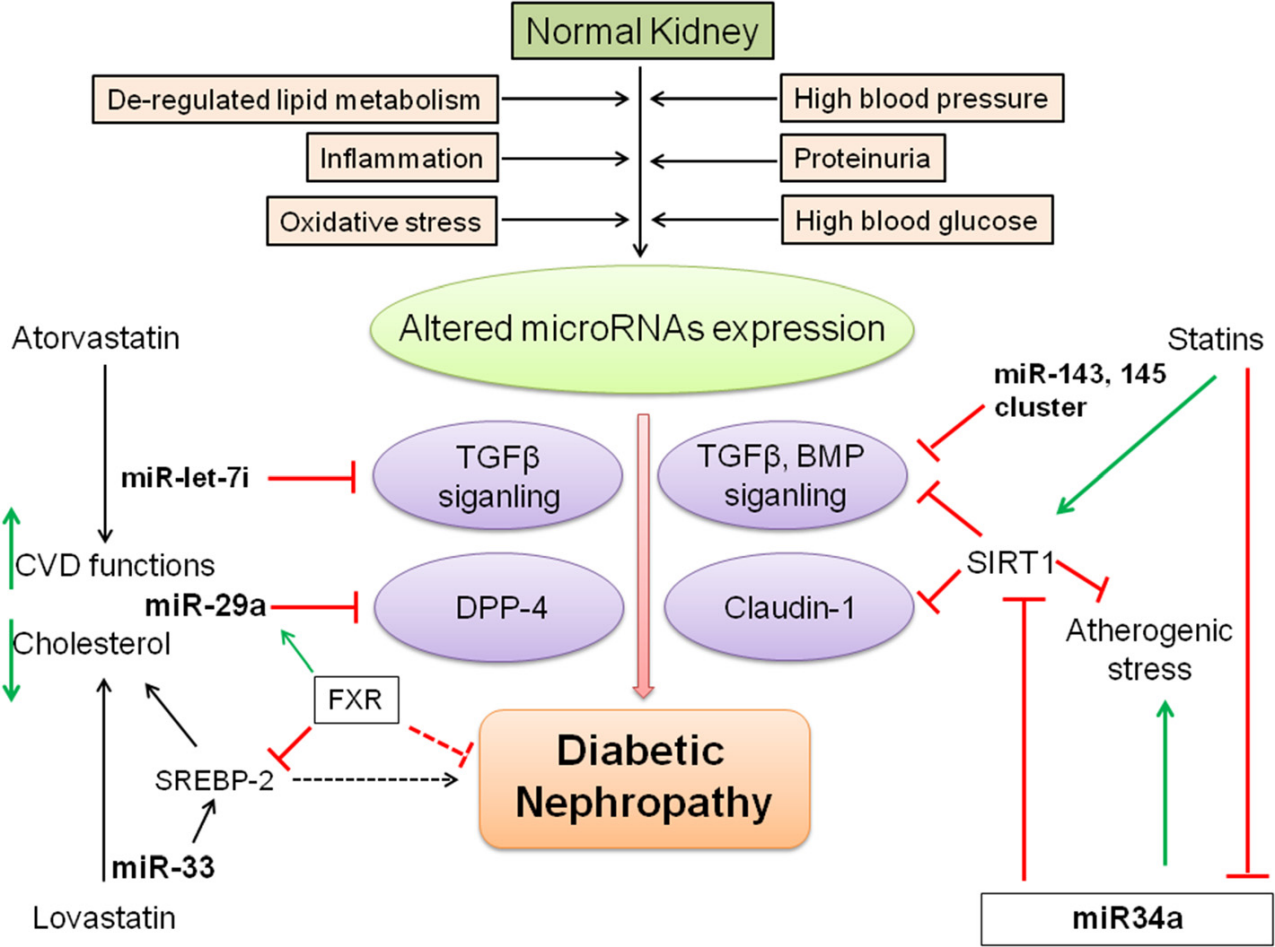
**Implications of antidyslipidemic drugs on the antifibrotic-miRs in the protective measures of DN.** Altered microRNA expression, as a result of a deregulated lipid metabolism, inflammation, oxidative stress, high blood pressure, proteinuria, and high blood glucose could cause DN by activating transforming growth factor β (TGFβ) signaling. An elevated DPP-4 level, BMP signaling, and claudin-1 could also be associated with DN. Atorvastatin improves cardiovascular functions by elevating miR-let-7i. miR-let-7i is associated with inhibiting TGFβ signaling. Farnesoid X receptor (FXR) is known to activate miR-29a, reducing DPP-4 and the associated renal fibrosis. Statins also elevate SIRT1 by inhibiting miR-34a. SIRT1 inhibits claudin-1 and associated renal fibrosis.

MicroRNAs play an important role in the pathogenesis and regulation of kidney diseases [62,63]. MicroRNAs are non-coding, single strands of 22 nucleotides that exert posttranscriptional control on gene expression by pairing their seed sequences (2 to 8 nucleotides at the 5′ end) to complementary sequences that are typically in the 3′ untranslated region of target mRNAs. This pairing results in the degradation of the target mRNA and/or the inhibition of its translation. Figure 3 depicts a summary of the microRNA regulation over antidyslipidaemic drugs and renal fibrosis. Statin treatment activates the expression of the miR-143/145 cluster through KLF2 (Kruppel-like family of transcription factor, the zinc finger family of DNA-binding transcription factor) in ECs [64]. MiR-143 and miR-145 are intergenic miRNAs that control the vascular smooth muscle cell (VSMC) phenotypic switch, tumorigenesis, and adipocyte differentiation [65]. ApoE knockout mice showed low levels of vascular miR-143/145 [66]. For instance, the miR-143 to 145 gene locus, which encodes both miR-143 and miR-145, is transcriptionally regulated by a complex composed of serum response factor (SRF) and myocardin or myocardin related transcription factors (MRTFs) [67,68]. TGF-β and BMP signaling increase miR-143 and miR-145 by activating the myocardin and MRTF, respectively [68]. Recently, atorvastatin was found to be associated with the upregulation of SIRT-1 expression via the inhibition of miR-34a [69]. The study elucidated a new mechanism by which statin therapy could improve endothelial dysfunction. miR-34a is an endogenous inhibitor of SIRT1, which promotes endothelial senescence. Renal tubular SIRT-1 attenuates diabetic albuminuria by epigenetically suppressing Claudin-1 overexpression in podocytes [70]. Atorvastatin improves CVD function by ameliorating miR-let7i level. Our research group recently suggested that the miR-let7s-FGFR1 axis inhibits TGFβ signaling [71]. Similarly, lovastatin ameliorates miR-33 family members, which in turn inhibit SREBP-2 and cholesterol synthesis. Farnesoid X Receptor (FXR) deficiency was found to cause diabetes to accelerate in type 1 DN mice [72]. FXR inhibits SREBP-2 and elevates miR-29a, thereby ameliorating renal fibrosis [73]. miR-29s have been shown to ameliorate renal fibrosis by downregulating the DPP-4 level [74]. The exact mechanisms by which FXR works to protect DN are not known, but elevating miR-29a could be one possibility [72].

### Perspective

The connection between atherosclerosis and glomerulosclerosis was suggested approximately 20 years ago by Diamond, who envisaged the foam cell, that is, the lipid overloaded macrophage, as the pivotal factor in both atherosclerosis and glomerulosclerosis [75,76]. Clinical studies suggest that lipid lowering drugs improve the cardiovascular functions in the DN patients, but no remarkable improvement has been found in the kidney outcome [4,5]. At the same time, clinical data revealed a reduction in the proteinuria in the statin-treated DN patients. Large-scale clinical trials that are prospective, randomized, and controlled are still lacking. Lipid control appears to be important in the prevention and treatment of DN. Targeting DAG over-production mediated PKC activation would allow for the utilization of cellular signaling pathways, which would be helpful for further studies on the control of DN. We previously discussed the role of ceramide and its effect on causing kidney disease. Targeting ceramide metabolism would be a therapeutic challenge in DN. Considering the microRNAs that target ceramide biosynthesis, degradation, and signaling mechanism, we could explore new insights and understandings into their role in the therapeutics of DN.

## Conclusion

The present review describes various aspects and implications of lipid lowering drugs in the treatment of DN. Enhanced PKC activation, as a result of hyperglycemia-mediated DAG production and an increased rate of ceramide synthesis, is associated with impairment in renal functions. Targeting PKC activation and ceramide metabolism would be a therapeutic challenge in DN. At the same time, treatment with lipid lowering drugs contributes to the upregulation of antifibrotic microRNAs and associated mechanisms, which would affect the therapeutic potential in DN.

## Abbreviations

ACR: Albumin to creatinine ratio; aPKC: atypical PKC; BP: Blood pressure; α-SMA: α-Smooth muscle actin; CARDS: Collaborative Atorvastatin Diabetes Study; CKD: Chronic kidney disease; cPKC: Conventional PKC; DN: Diabetic nephropathy; DAG: Diacyglycerol; EPA: Eicosapentaenoic acid; ECM: Extracellular matrix protein; eGFR: Estimated glomerular filtration rate; FIELD: Fenofibrate Intervention and Event Lowering in Diabetes; FXR: Farnesoid X Receptor; HDL-c: High density lipoprotein cholesterol; HMG CoA: 3-hydroxy-3-methyl-glutaryl-CoA; JAS: Japanese atherosclerosis society; KLF2: Kruppel-like family of transcription factor; LDL-c: Low-density lipoprotein cholesterol; MRTFs: Myocardin related transcription factors; MAPK: Mitogen activated protein kinase; MCP-1: Monocyte chemotactic protein-1; NO: Nitric oxide; nPKC: New PKC; PANDA: Protection Against Nephropathy in Diabetes with Atorvastatin; PKCs: Protein kinase C; PAI-1: Plasminogen activator inhibitor-1; PS: Phosphatidylserine; PE: Phorbolester; PPARα: Peroxisome proliferator-activated receptor; PRK: PKC related kinases; PHN: Passive Heymann nephritis; ROS: Reactive oxygen species; SIRT1: Sirtuin1; SHARP: Study of Heart and Renal Protection; SRF: Serum response factor; SERBP: Sterol-Regulator Element-Binding Proteins; TGFβ1: Transforming growth factor β1; TXA2: Thromboxane A2; VEGF: Vascular endothelial growth factor; VSMCs: Vascular smooth muscle cells.

## Competing interests

The authors declare no competing interest in this work.

## Authors’ contributions

SPS designed the study, wrote the review manuscript, and was involved in the discussion. SS was mainly involved in the editing process. DK made intellectual contributions. KK conceived of the project, provided intellectual contribution, and guided the manuscript writing and editing. All authors read and approved of the final manuscript.
